# KIR4.1: K^+^ Channel Illusion or Reality in the Autoimmune Pathogenesis of Multiple Sclerosis

**DOI:** 10.3389/fnmol.2016.00090

**Published:** 2016-09-27

**Authors:** Chen Gu

**Affiliations:** Department of Biological Chemistry and Pharmacology, The Ohio State UniversityColumbus, OH, USA

**Keywords:** multiple sclerosis, KIR4.1 channel, autoantibody, aquaporin 4, astrocyte, myelin

## Abstract

Multiple sclerosis (MS) is an inflammatory demyelinating disease of the central nervous system (CNS). Many believe autoimmune pathogenesis plays a key role in MS, but its target(s) remains elusive. A recent study detected autoantibodies against KIR4.1, an ATP-sensitive, inward rectifier potassium channel, in nearly half of the MS patients examined. KIR4.1 channels are expressed in astrocytes. Together with aquaporin 4 (AQP4) water channels, they regulate astrocytic functions vital for myelination. Autoantibodies against AQP4 have been established as a key biomarker for neuromyelitis optica (NMO) and contributed to diagnostic and treatment strategy adjustments. Similarly, identification of KIR4.1 autoantibodies could have high therapeutic values in treating MS. Consistent with its potential role in MS, KIR4.1 dysfunction is implicated in several neurological disorders. However, the enrichment of KIR4.1 autoantibodies in MS patients is questioned by follow-up studies. Further, investigations are needed to clarify this controversy and unravel the underlying mechanisms of MS pathogenesis.

MS affects the brain and spinal cord of over two million people worldwide. The primary MS pathology includes immune-mediated destruction of myelin sheaths and axon degeneration. Early MS symptoms include fatigue, walking difficulties, blurred vision, and numbness, tingling, and weakness of the limbs or other parts of the body. Others include muscle stiffness, cognitive deficits, and urinary problems. The cause (etiology) of MS is still unknown, but it is believed several interacting factors are involved, such as genetic, environmental, infectious, and most importantly, immunological factors. Antibody-generating B cells, as well as cytotoxic T cells, are most likely involved in the abnormal autoimmune attack of myelin and axons common in MS. The importance of B cells in the autoimmune pathogenesis of MS is supported by increased immunoglobulins in the cerebrospinal fluid (CSF) and demyelinated lesions in the CNS of MS patients, as well as by the B-cell-depleting monoclonal antibody, rituximab, which decreases MS relapses (Hauser et al., 2008). However, although specific autoantibodies in MS patients have been targets of intensive investigation for decades, the identification of autoantigen(s) in MS still remains elusive. The discovery of potential autoantigen in MS often gives rise to controversy. KIR4.1 channel was recently identified to play a key role in the MS pathogenesis (Srivastava et al., 2012), but has debatable potential as a candidate autoantigen.

KIR channels (KIR1 to KIR7) are inwardly rectifying channels, which have a greater tendency to allow K^+^ ions to flow into, rather than out of, a cell. These KIR channels are not sensitive to dalfampridine [4-aminopyridine (4-AP)], which is currently used for symptomatic treatment of MS, particularly for improving walking in MS patients [see reviews for detailed information (Judge and Bever, 2006; Blight et al., 2014)]. It is believed that the beneficial effect of 4-AP results from restoring axonal conduction by blocking voltage-gated K^+^ (Kv) channels that are exposed in demyelinated axons. However, 4-AP does not cure MS and blocks many types of Kv channels that are broadly expressed in the nervous system. KIR channels are different from Kv channels in terms of both structure and function. The KIR α-subunit contains two transmembrane domains and one pore-forming loop (Figure 1A). Four KIR α-subunits homo- or hetero-tetramerize to assemble into a functional channel. KIR channels are also insensitive to another Kv channel blocker tetraethylammonium, but can be blocked by Ba^2+^. Among about 80 genes in the K^+^ channel superfamily, 15 KIR α-subunits have been identified in humans and rodents with different biophysical and pharmacological properties, and patterns of tissue expression and subcellular localization. KIR4.1 is an intermediate inward rectifier and can bind to adenosine triphosphate (ATP) and PtdIns(4,5)P_2_ (Hibino et al., 2010).

**Figure 1:**
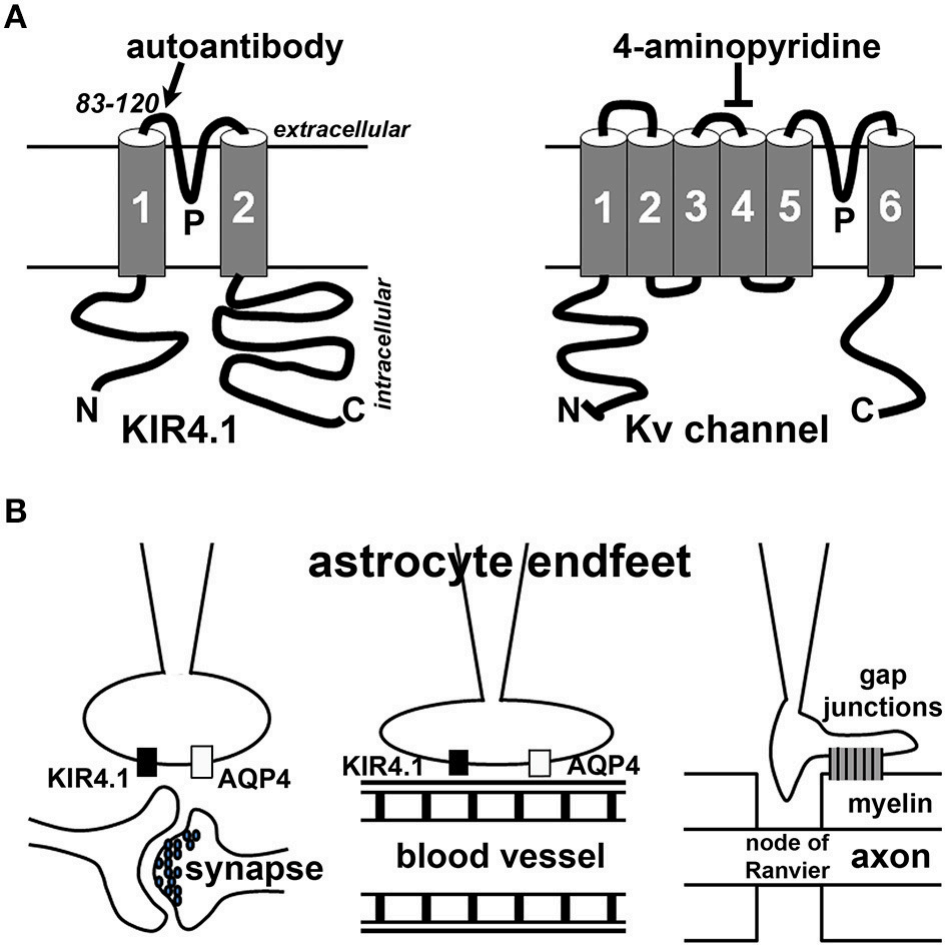
**Schematic diagrams of KIR4.1 structure and localization in astrocyte endfeet**. **(A)** Structural diagrams of KIR4.1 (left) and Kv channel (right) subunits. N, the N-terminus; C, the C-terminus; P, the P-loop. The region near the P-loop (residues 83–120) is the putative site recognized by the autoantibodies (Srivastava et al., 2012). 4-Aminopyridine blocks some Kv channels but not KIR channels. **(B)** Astrocyte endfeet interact with synapses (left), blood vessels (middle) or myelin membranes (right).

KIR4.1 channels are expressed in the CNS and usually localize in astrocytic endfeet (Figure 1B). These channels play an important “K^+^ buffering” role, removing K^+^ ions from extracellular spaces surrounding astrocytes adjacent to blood vessels and synapses. KIR4.1 reduction can cause neuronal dysfunction and seizure activity in animals. Globally deleting KIR4.1 in mice causes impaired myelination and oligodendrocyte maturation during development, leading to neuronal degeneration and mice surviving only until around 2–3 weeks of age (Neusch et al., 2001). Mice with a KIR4.1 genetic polymorphism display increased seizure susceptibility, likely resulting from impaired astrocytic K^+^ and glutamate uptake (Inyushin et al., 2010). Conditionally deleting KIR4.1 from GFAP (glial fibrillary acidic protein)-positive astrocytes in mice also caused impaired K^+^ and glutamate uptake, leading to ataxia, seizures, and premature death (Djukic et al., 2007). Therefore, KIR4.1 is critical for the proper functions of astrocytes vital for the CNS.

Although oligodendrocytes and T lymphocytes are the main focus of MS research, there has been a surge of interest in studying astrocytes in the pathogenic process of MS. Astrocytes are a diverse group of non-neuronal cells and the most abundant cell type in the brain. They can be divided into three major types: protoplasmic astrocytes (in gray matter), fibrous astrocytes (in white matter), and radial glial cells. Through their endfeet, astrocytes regulate synaptic transmission, blood-brain barrier, and myelination (Figure 1B). Astrocytic dysfunction has also been implicated in Alzheimer's disease, Huntington disease, epilepsy, stroke, and a variety of other neurological disorders and conditions (see a recent review, Pekny et al., 2016). In MS, astrocytes can regulate the pathogenesis by acting as part of the innate immune system, releasing soluble factors including trophic factors to promote remyelination, forming a glial scar to inhibit remyelination and axonal regeneration, and maintaining the proper function of the neurovascular unit [see recent reviews for detailed information (Brosnan and Raine, 2013; Nualart-Marti et al., 2013; Ludwin et al., 2016). More extensive discussion of astrocytes in MS and neurological disorders is beyond the scope of this review article.

KIR4.1 channel has been linked to demyelinating conditions. By screening serum IgG from MS patients, Srivastava et al. recently identified IgG1 and IgG3 antibodies that can specifically bind to glial cells in the CNS of humans and rats (Srivastava et al., 2012). Using enriched IgG fraction, they immunoprecipitated lysates of human brain tissue and identified precipitated proteins with tandem mass spectrometry. One of the identified proteins was KIR4.1. A series of assays including enzyme-linked immunosorbent assay (ELISA) and Western blotting were used to confirm KIR4.1 as a target of the autoantibody response in MS (Srivastava et al., 2012). Importantly, the study showed that serum levels of antibodies to KIR4.1 were higher in MS patients (about 47% of 397) than persons with other neurologic diseases (about 1% of 329) and healthy donors (0% of 59). These antibodies appeared to bind to the peptide (residues 83–120) corresponding to the first extracellular loop of KIR4.1, and thus can bind to the channel without entering the cell. Furthermore, injection of KIR4.1 serum IgG into the cisternae magnae of mice caused a profound loss of KIR4.1 expression, altered expression of GFAP in astrocytes, and activation of the complement cascade at sites of KIR4.1 expression in the cerebellum. Based on these results, the authors proposed that KIR4.1 is an autoimmune target in some MS patients (Srivastava et al., 2012). Later, Krau et al. from the same research group further showed that serum antibodies to KIR4.1 were present in more than half of children with acquired demyelinating disease but not in children with other diseases or in healthy controls (Kraus et al., 2014). This study suggests that KIR4.1 is also an important target of autoantibodies in childhood acquired demyelinating disease.

The KIR4.1 study in MS autoimmune pathogenesis drew an interesting comparison to the role of a water channel, AQP4, as an important autoimmune target in another inflammatory demyelinating disease, NMO (Lennon et al., 2004; Waters and Vincent, 2008; Hinson et al., 2012). NMO was thought to be one form of MS until the discovery of the autoantibody in NMO suggesting different mechanisms governing NMO and MS pathogeneses. Different from MS, NMO is a primary astrocytopathy with secondary demyelination. MS and NMO also differ in pathological and imaging features, as well as in their responses to different immunotherapies (see recent reviews, Barnett and Sutton, 2012; Jukkola and Gu, 2015). In the CNS, AQP4 is predominantly localized to astrocytic endfeet contacting blood capillaries and synapses, similar to KIR4.1 (Figure 1B). The colocalization led to the hypothesis that the two channels functionally interact to regulate water and K^+^ homeostasis. However, they unlikely interact directly, since KIR4.1 channel expression and function remain unchanged in AQP4 knockout mice, and inhibition or knockdown of KIR4.1 does not change the AQP4 water permeability (Ruiz-Ederra et al., 2007; Zhang and Verkman, 2008). Nonetheless, besides direct regulation of water homeostasis, AQP4 can indirectly regulate K^+^ homeostasis through its effect on extracellular space volume, for instance during synaptic stimulation (Haj-Yasein et al., 2015). It is important to note that APQ4 autoantibody was present in only about 65% of NMO patients, and completely absent in the serum of MS patients, whereas KIR4.1 autoantibody was suggested to be present in about half of MS patients (Srivastava et al., 2012). Therefore, it seems that additional targets of autoantibodies must be present in both NMO and MS.

Mutations in KIR4.1 have been linked to epilepsy, ataxia and deafness in in humans (Bockenhauer et al., 2009). Hippocampal sclerosis in temporal lobe epilepsy appears to be associated with loss of KIR4.1 in astrocytic endfeet (Heuser et al., 2012). In animal models of epilepsy, disruption of the blood brain barrier led to serum albumin leakage into the CNS and uptake by astrocytes, leading to downregulation of KIR4.1 and the development of neuronal hyperexcitability and epileptic activity (Cacheaux et al., 2009). Reduced KIR4.1 activity causes increased extracellular K^+^ and glutamate, contributing to brain hyperexcitability and epileptogenesis. Furthermore, deficits of astrocytic KIR4.1 channel may contribute to neuronal dysfunction in the mouse model of Huntington's disease (Tong et al., 2014). As a result of KIR4.1 reduction, extracellular K^+^ levels measured by K^+^-selective microelectrodes were elevated from 1.5 mM (under control condition) to about 3.0 mM, which significantly depolarized striatal medium spiny neurons by ~6 mV (e.g., from −75 to −69 mV) and increased their excitability. This can be rescued by over-expressing functional KIR4.1, leading to ameliorated deficits associated with the mouse models (Tong et al., 2014). A spinal cord injury model showed up to 80% reduction in Kir4.1 expression in the lesion area, and demonstrated that a previously discovered neuroprotective agent, 17-β-oestradiol, could partially restore KIR4.1 expression and function (Olsen et al., 2010).

Although KIR4.1 channel appears to be a plausible target of the antibody response in MS pathogenesis, the subsequent and independent studies have provided inconsistent results and casted doubt regarding whether there is indeed an increase of KIR4.1 autoantibodies in MS patients. Using ELISA with synthetic human KIR4.1 peptide (residue 83–120), Nerrant et al. found KIR4.1 autoantibodies in serum from only 7.5% of MS patients (total 268), 4.3% of patients with other neurological diseases (total 46) and 4.4% of healthy controls (total 45; Nerrant et al., 2014). Using the same peptide-based ELISA and immunostaining, Brickshawana et al. failed to detect KIR4.1-specific IgG in serum or CSF from MS patients, or any KIR4.1 loss from glia in MS lesions (Brickshawana et al., 2014). In this study, the investigators tested 229 population-based and 57 clinic-based MS patients, 99 healthy controls, and 109 disease controls (Brickshawana et al., 2014). On the other hand, using the peptide-based ELISA, Brill et al. identified KIR4.1 autoantibodies in serum from 21 of 80 MS patients, 10 of 45 NMO patients, and 2 of 32 healthy controls (Brill et al., 2015). Interestingly, KIR4.1 antibody levels appeared to be higher during relapse than remission in MS patients, so the antibody may represent a marker of disease exacerbation (Brill et al., 2015). In these three more recent and relatively negative studies, the peptide-based (KIR4.1 residue 83–120) ELISA was used, whereas the whole-KIR4.1-based ELISA was used in the prior two positive studies from the same group (Srivastava et al., 2012; Kraus et al., 2014). The channel peptide may not have the same conformation as in a native channel complex to be recognized by the antibodies. Moreover, glycosylation present in the native channel is not reflected in the peptide. For instance, within the first extracellular loop (83–120) of KIR4.1, there is a potential N-linked glycosylation site at N104 (NHT). Therefore, it is possible that antibodies bind to the whole channel but not to the synthetic peptide of the binding site.

In order to resolve the controversy regarding whether KIR4.1 autoantibodies are present in MS patients, two additional studies from independent groups performed the whole-KIR4.1-based ELISA and published their results in the New England Journal of Medicine in 2016. In the first study, the full-length KIR4.1 channel protein was expressed in HEK293 cells and purified. This procedure allowed for KIR4.1 native tetrameric assembly and isolation of low-glycosylated KIR4.1 isoforms, which are critical for autoantibody binding. Chastre et al. performed the assay using detailed instructions provided by the authors of the original report during a visit to their laboratory as part of a collaborative scientific exchange. After testing serum from 86 clinic-based MS patients and 51 healthy control donors, none of the samples showed KIR4.1 reactivity and no significant difference was established between MS patient and control group (Chastre et al., 2016). Due to technical difficulties including the impact of high-order structure formation of the KIR4.1 channel complex and post translational modifications on KIR4.1-antibody binding, Chastre et al. concluded that further studies are required to clarify the presence of KIR4.1 autoantibodies in MS, including investigations through cooperative sharing of specimens (Chastre et al., 2016).

In the second study, Probstel et al. performed a large, blinded study testing serum samples from 141 MS patients and 131 controls, with both a protein and a peptide ELISA, and failed to detect a significant difference between MS and control groups in KIR4.1 autoantibodies (Probstel et al., 2016). This study further suggested that the whole-protein ELISA might reveal serum reactivity directed against non-KIR4.1 proteins copurified with KIR4.1 (Probstel et al., 2016). Despite the negative result, the authors also cautioned the specificity of ELISA technique for detecting KIR4.1 autoantibodies and suggested further investigation (Probstel et al., 2016). Taken together, KIR4.1 channel cannot currently be established as an important autoimmune target in MS pathogenesis.

Another recent study failed to detect enriched KIR4.1 autoantibodies in Japanese patents with MS (57 patients) or NMO (40 patients including NMO spectrum disorders), compared to 50 healthy controls (all were Japanese; Higuchi et al., 2016). ELISA using a synthetic peptide of the first extracellular portion of human KIR4.1 (residues 83–120) did not reveal even a single positive in serum samples from those patients. Antibodies against the full length KIR4.1 were detected in only two MS patients and in none of NMO patients (Higuchi et al., 2016). Nonetheless, since anti-KIR4.1 autoantibodies were detected at a very low frequency in MS patients, the authors suggest that at least currently serum testing for human KIR4.1-specific antibodies is unlikely to improve the diagnosis of MS in Japanese patients.

Regardless of whether KIR4.1 autoantibodies are enriched in MS patients, further investigations are still needed to identify the target(s) of the antibody response in MS, as well as in NMO. Even in NMO, AQP4 autoantibodies are not present in every patient, suggesting the presence of other target(s). To unequivocally identify the target(s) of MS autoantibodies, future investigations need cooperative studies using shared serum samples from MS patients and healthy controls, as well as established detection procedures and reagents. However, it is perhaps more important to clarify first whether the autoantibodies are pathogenic or only byproducts from degenerative process of astrocytes in the CNS during disease progression. For instance, it is not yet clear whether autoantibodies have to bind the extracellular portion of the channel, or can be first internalized and subsequently bind to intracellular portion of the channel. How antibodies alter channel functions still remains unknown. Furthermore, astrocytic KIR4.1 channel does not likely operate alone. Its binding proteins and other proteins involved in regulating astrocytic functions, such as KIR5.1 and AQP4, could also be the target causing similar disruption, and are worth of pursuing. Indeed, many ion channel proteins have been identified to express in resting or activated astrocytes (Jukkola and Gu, 2015). They include Kv channels, two-pore K^+^ channels, several voltage-gated Ca^2+^ and Na^+^ channels, transient receptor potential channels, and gap junctions (Jukkola and Gu, 2015). Their disruption also likely causes dysfunction of astrocytic signaling, leading to CNS malfunction. In sum, further investigation in this field will contribute to a better understanding of the immune pathogenesis of MS, as well as the development of new treatments for autoimmune diseases.

## Author contributions

The author confirms being the sole contributor of this work and approved it for publication.

### Conflict of interest statement

The author declares that the research was conducted in the absence of any commercial or financial relationships that could be construed as a potential conflict of interest.
